# Dapsone and schistocytes: Thrombotic microangiopathy or not?

**DOI:** 10.1371/journal.pone.0349570

**Published:** 2026-05-22

**Authors:** Alexis Archambeaud, Cédric Aumont, Claire Pouplard, Marie-Sarah Agier, François Maillot, Jean-Michel Halimi, Adrien Bigot

**Affiliations:** 1 Department of Internal Medicine, CHU Tours, Tours, France; 2 Department of Haematology, CHU Tours, Tours, France; 3 University of Tours, INSERM ISCHEMIA U 1327, Tours, France; 4 University of Tours, University of Nantes, INSERM 1246-SPHERE, Tours; CHU Tours, Regional Pharmacovigilance Centre, Tours, France; 5 Department of Nephrology, CHU Tours, Tours, France; University of Sindh, PAKISTAN

## Abstract

**Introduction:**

Dapsone is used in many infectious and immunological diseases. Dapsone may cause haemolysis, particularly in individuals with glucose-6-phosphate dehydrogenase (G6PD) deficiency. This hemolysis is described as oxidative and is usually not associated with the presence of schistocytes.

**Patients and methods:**

We conducted a single-centre retrospective observational study at the University Hospital of Tours, France (2005–2021). Adult patients who received dapsone and had a blood smear performed during treatment or within 7 days of discontinuation were included. Data were extracted from electronic medical records. Patients were classified as schistocyte-positive (≥0.1%) or -negative. Descriptive statistics were used; no further analyses were performed due to small sample size.

**Results:**

During the 2005−2021 period, 19 adult patients received dapsone in our institution. Amongst them, 10 (52.6%) had detectable schistocytes, ranging from 2 to 44/1000 erythrocytes (13 ± 14.2). Patients with schistocytosis exhibited more severe hemolytic anemia, with a larger decrease in hemoglobin (−2.7 ± 1.4 g/dL vs −1.6 ± 2.12 g/dL), higher methemoglobinemia (5.2% vs 1.9%), more frequent low haptoglobin levels (88.9% vs 60%), and elevated LDH levels (557 U/L [239–671] vs 273 U/L [208–507]) compared to patients without schistocytosis. Platelet counts were similar between the two groups. No organ failure was identified in the reviewed medical records. Schistocytosis regressed upon discontinuation of treatment after 3–18 days.

**Conclusion:**

Dapsone appears to be associated with hemolytic anemia and the presence of schistocytes, suggesting mechanical intravascular haemolytic anaemia. Whether Dapsone-associated biological thrombotic microangiopathy can be associated with target organ damage requires further investigation.

## Introduction

Dapsone (4,4’-diaminodiphenylsulfone) has been used to treat leprosy since the 1950s and its clinical applications have expanded over time [1]. It is currently used in several diseases including autoimmune and neutrophilic dermatoses, bullous lupus, pneumocystis prophylaxis, relapsing polychondritis and immune thrombocytopenia (ITP).

Haemolysis, a well-known side effect of dapsone [2–4], results from the oxidative denaturation of haemoglobin [5,6]. Several cytological abnormalities may be associated, including Heinz bodies [7–9] and bite cells [10,11]. In contrast, schistocytes have rarely been reported with dapsone treatment [12,13], including in patients with G6PD deficiency [14]. However, the characteristics of patients treated with dapsone who developed schistocytosis are presently unknown. The presence of schistocytes in the context of anemia may indicate mechanical hemolysis, reflecting microangiopathic hemolytic anemia, which is the hallmark of thrombotic microangiopathy (TMAs). TMAs comprise a heterogeneous group of disorders characterized by microangiopathic hemolytic anemia, thrombocytopenia, and microvascular thrombosis leading to ischemic tissue injury, most commonly involving the kidneys [15]. Although TMAs have historically been diagnosed on the basis of the classical clinical triad without systematic pathological confirmation, recent studies indicate that a substantial proportion of patients with biopsy-proven TMA do not exhibit significant hematologic abnormalities, supporting the existence of a kidney-limited phenotype [16]. Consistently, the classical triad—defined by thrombocytopenia, anemia, and elevated lactate dehydrogenase—is absent in more than 40% of cases across the spectrum of TMA etiologies [17]. Dapsone is not currently recognized as a causative agent of TMA [18]. However, the observation of anemia and thrombocytopenia with schistocytes in several patients treated with dapsone at our institution prompted us to consider whether this association could reflect a possible thrombotic microangiopathy. In this retrospective study, we assessed the clinical and biological characteristics associated with schistocytosis among patients treated with dapsone.

## Patients and methods

### Ethics approval

As part of this retrospective study based on medical records and archived samples, written informed consent was obtained freely from all participants (information letter with an objection form, including the investigator’s contact details for further explanations. According to current French regulations, a registration was made with the CNIL (National Commission for Information Technology and Civil Liberties). A record was made in the data processing register of the University Hospital of Tours (number 2022_086). All data were anonymized prior to access using a unique registration code within the institution.

### Study design and data collection

This was a single-centre retrospective observational study, conducted at the University Hospital of Tours in France from January 2005 to December 2021. Epidemiological, demographic, clinical, laboratory, and treatment data were extracted from electronic medical files. We searched the hospital database for all patient medical records with the keyword “dapsone” from January 2005 to December 2021. We cross-referenced the results with computer codes to identify every blood smear performed in the hospital. Each medical record was reviewed to ensure that patients were prescribed dapsone. Blood smears performed during treatment or within 7 days of dapsone discontinuation were collected. If schistocytes were detected, the results of previous and subsequent smears were analysed to evaluate the course after treatment discontinuation.

The data were accessed for research purposes starting on July 22, 2022. Only the first author initially had access to information allowing individual identification of the participants during or after data collection.

### Study setting and population

The study population included patients over 18 years old who had a blood smear performed while they were being treated with dapsone or within 7 days following discontinuation. Each blood smear was reviewed by a trained haematologist, specifically for this study, blinded to the original results previously reported and to the clinical data. For each patient, the search for schistocytes was performed on a peripheral blood sample with microscopic investigation on blood smear after May Grunwald Giemsa staining.

### Statistical analysis

Demographic characteristics, baseline clinical characteristics, and laboratory values were summarized using descriptive statistics. Patients were divided into two groups based on blood smear findings: Group A (presence of schistocytes) and Group B (absence), using a threshold of 0.1% to define positivity.

No statistical analyses were performed due to the small number of patients included in the study; all data are presented descriptively.

## Results

### Identification of patients

**Fig 1:**
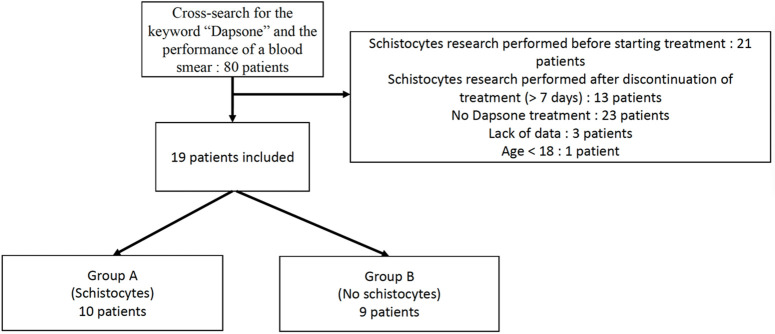
Study flow diagram.

The cross-search in the local database for the keyword “dapsone” and the performance of a blood smear identified 80 patients. Following manual file review, 61 patients were excluded due to age (age under 18 years), missing data, not having taken dapsone, or not having a blood smear during treatment or within 7 days of discontinuation. Hence, 19 patients were included in the study (Fig 1).

Thirty-four blood smears were identified, of which 22 from 19 patients could be retrieved for review and 12 were unavailable. All available blood smears were stored at the University Hospital of Tours. A hematologist re-evaluated all remaining blood smears, confirming the initial findings for both the presence or absence of abnormalities and the quantitative assessment. Two groups were created — Group A (n = 10) with schistocytes and Group B (n = 9) without.

### Patient characteristics

Epidemiological, demographic, clinical, laboratory, and treatment data are reported in Table 1. Patients were predominantly male (11/19 patients). They were older in group A (64.7 years [33–84]) than in group B (59.6 years [18–78]). The indication for treatment with dapsone was mainly ITP (13/19 patients). The median dose was 100 mg per day in each group. The median time since the start of treatment was similar in both groups. Some blood smears were performed after discontinuation of treatment (within 7 days after discontinuation) in 4 patients of group A. Four patients of group A had blood smears prior to dapsone treatment, none of which had schistocytes. Among associated treatments, simvastatin (1 patient) and progesterone (1 patient) were found in group A, and simvastatin (1 patient), metronidazole (1 patient) and cotrimoxazole (1 patient) in group B. No patient had a mechanical heart valve. A haematological malignancy was observed in 1 patient of group A (stage 4 follicular lymphoma). A history of splenectomy was observed in 1 patient of group B. One patient in group A had suspicious pulmonary nodules at the time of the blood smear (subsequent investigations revealed pulmonary metastases). No obvious cause of TMA was observed (no pregnancy, HIV infection, antiphospholipid syndrome, solid organ transplantation or haematopoietic stem cell transplantation). One patient in each of groups A and B had a non-severe infection at the time of the blood smear (prostatitis and legionellosis respectively). Several bleedings were reported in patients followed for ITP: 4 patients in group A (median platelet count: 39 G/L) and 1 patient in group B (median platelet count: 60 G/L).

**Table 1:** Demographic and clinical characteristics at baseline.

Characteristics	Group A: schistocytes (n = 10)	Group B: no schistocytes (n = 9)
Age (range), years	64.7 (33-84)	59.6 (18-78)
Female sex, no.	2	6
Pathology, no.		
ITP	6	7
Pemphigoid	3	2
Others	1	0
Dapsone dosage, no.		
25 mg	0	1
50 mg	1	2
100 mg	7	6
150 mg	1	0
200 mg	1	0
Time elapsed since the beginning of treatment (range), days	53 (15-407)	57 (7-2270)
Comorbidities, no.		
Hypertension	5	3
Diabetes	3	0
Heart arrhythmia	1	2
Coronary heart disease	2	2
Valvular prosthesis	1	1
Vascular prosthesis	1	0
Haematological malignancy	1	0
Sarcoidosis	0	1
Lupus	1	2
Multiple sclerosis	1	0
Splenectomy	0	1
Active cancer	1	0
Others: HIV infection, ongoing pregnancy, haematopoietic stem cell transplantation, solid organ transplant, haemoglobinopathy	0	0
Infectious diseases, no.	1	1

data are presented as number or median (range).

Schistocyte discovery was fortuitous in all cases and no target organ damage was observed, despite extensive investigations in patients with suspected TMA. At the least, patients with ITP were hospitalised or had prolonged hospitalisation due to the discovery of schistocytosis and suspected primary TMA.

### Haemolysis biological parameters

The median schistocyte rate in group A was 1.3% (0.2–4.4). Compared to group B (Table 2), group A had more severe anaemia (haemoglobin: 10.6g/dL [7.8–12.6] vs 12.7g/dL [6.9–14.1]), a more pronounced decrease in haemoglobin after introduction of treatment (−2.7g/dL [−0.4; −5.4] vs −1.6g/dL [−0.4; −7.4]), higher reticulocyte count (189 G/L [66–306] vs 126 G/L [35–300]), increased mean corpuscular volume (98.8fL [87.9–110.5] vs 93.3fL [85.1–97.5]), elevated lactate dehydrogenase (557U/L [239–671] vs 273U/L [208–507]), and more frequently decreased haptoglobin (8/9 vs 3/5). Free bilirubin levels were similar between the 2 groups. Platelet count was low in several patients but was difficult to interpret due to many patients with a history of ITP. Among non-ITP patients, the platelet count was 216,000/mm^3^ (128–264) and 245,000/mm^3^ (187–304) respectively in groups A and B. The methaemoglobin level was higher in group A (5.2% [1.3–15.6]) vs 1.9% [1.4–2.4]). Serum creatinine following dapsone introduction was stable in the 2 groups (+ 2.5 μmol/L [−30; + 13] in group A and – 4.0 μmol/L [−24; + 18] in group B).

**Table 2:** Laboratory findings.

	Group A: schistocytes (n = 10)	Group B: no schistocytes (n = 9)
Schistocytes level, %	1.3 (0.2-4.4)	0
Haemoglobin, g/dL	10.6 (7.8-12.6)	12.7 (6.9-14.1)
Haemoglobin diff, g/dL	−2.7 (−0.4; −5.4)	−1.6 (−0.4; −7.4)
MCV, fL	98.8 (87.9-110.5)	93.3 (85.1-97.5)
Reticulocyte count, G/L	189 (66-306)^1^	126 (35-300)^2^
Platelet count, G/L	134 (7-330)	133 (29-304)
Platelet count diff, G/L	+20 (−70; + 325)	−17 (−46; + 110)
Leucocyte count, G/L	7.6 (3.2-31.9)	5.6 (3.8-13.3)
Leucocyte count diff, G/L	−0.7 (−4.3; + 23.2)	−2.1 (−6.2; + 0.8)
Creatinine, μmol/L	81 (55-166)^1^	75 (57-84)^3^
Creatinine diff, μmol/L	+2.5 (−30; + 13)^2^	−4.0 (−24; + 18)^4^
Estimated GFR, mL/min.1,73m^2^	85 (37-137)^1^	88 (58-122)^3^
GFR diff, mL/min.1,73m^2^	−5.0 (−18; + 48)^2^	+4 (−32; + 30)^4^
Lactate dehydrogenase, U/L	557 (239-671)^1^	273 (208-507)^5^
Unconjugated bilirubin, μmol/L	11 (8-18)^2^	9 (1-26)^4^
Haptoglobin < 0,3 g/L, no	8/9 (88.9%)^1^	3/5 (60%)^4^
Methaemoglobin level, %	5.2 (1.3-15.6)^2^	1.9 (1.4-2.4)^6^
CRP, mg/L	1.9 (0.4-51.4)^1^	3.7 (0.5-19.5)^4^
Vitamin B9 deficiency, no *	1^3^	2^5^
Vitamin B12 deficiency, no *	0^3^	0^5^
Iron deficiency, no **	0^3^	0^6^
Hypothyroidism, no ***	0^5^	0^4^
Prothrombin time < 70%, no	1^2^	2^5^
Activated partial thromboplastin time ratio > 1,20, no	1^3^	1^5^
Fibrinogen, g/L	3.2 (2.3-5.9)^3^	5.3 (4.4-6.2)^7^
Haemoglobin > 1 month after discontinuing treatment, g/dL	12.6 (10.5-14.6)^2^	/
Haemoglobin > 1 month after discontinuing treatment diff ^+^ , g/dL	+2.4 (+1.4; + 3.5)^2^	/
GFR > 1 month after discontinuing treatment diff, mL/min.1,73m^2^	+10 (−10; + 18)^2^	/

data are presented as number or median (range).

n1234567: 1,2,3,4,5, 6 or 7 missing data.

MCV: mean corpuscular volume; CRP: C-reactive protein; TSH: thyroid-stimulating hormone.

diff: difference between blood smear value and pre-treatment value.

diff + : difference between value > 1 month and blood smear value.

*: Vitamin deficiencies are defined by a vitamin B9 level < 8.8nmol/L and a vitamin B12 level < 145pmol/L.

**: Iron deficiency is defined by a ferritin level < 30ug/l or < 70ug/l when inflammation is present.

***: Hypothyroidism is exclusively defined by TSH values. Laboratory threshold: 0.4mUI/L < TSH < 6.3mUI/L.

ADAMTS 13 testing was performed on 4 patients in group A, and no significant deficit was observed. The vitamin assays showed a vitamin B9 deficiency in 1 patient in group A and 2 patients in group B, but neither iron nor vitamin B12 deficiency. In group A, a single patient had low prothrombin and elevated aPTT in the context of anti-vitamin K treatment. No decrease in fibrinogen levels was found in either group.

Glucose-6-phosphate dehydrogenase was tested in 7 patients and was found to be marginally decreased — to 111 U/gHb in one patient from group A and to 95 U/gHb in one patient from group B (laboratory threshold: 120 U/gHb), though not reaching the threshold for clinical G6PD deficiency. No unusual neurological signs or thrombotic organ damage were found, despite extensive investigations in some patients.

Detailed data for each patient in group A are provided in S1 Table.

### Progression of haematological parameters after dapsone discontinuation

**Fig 2:**
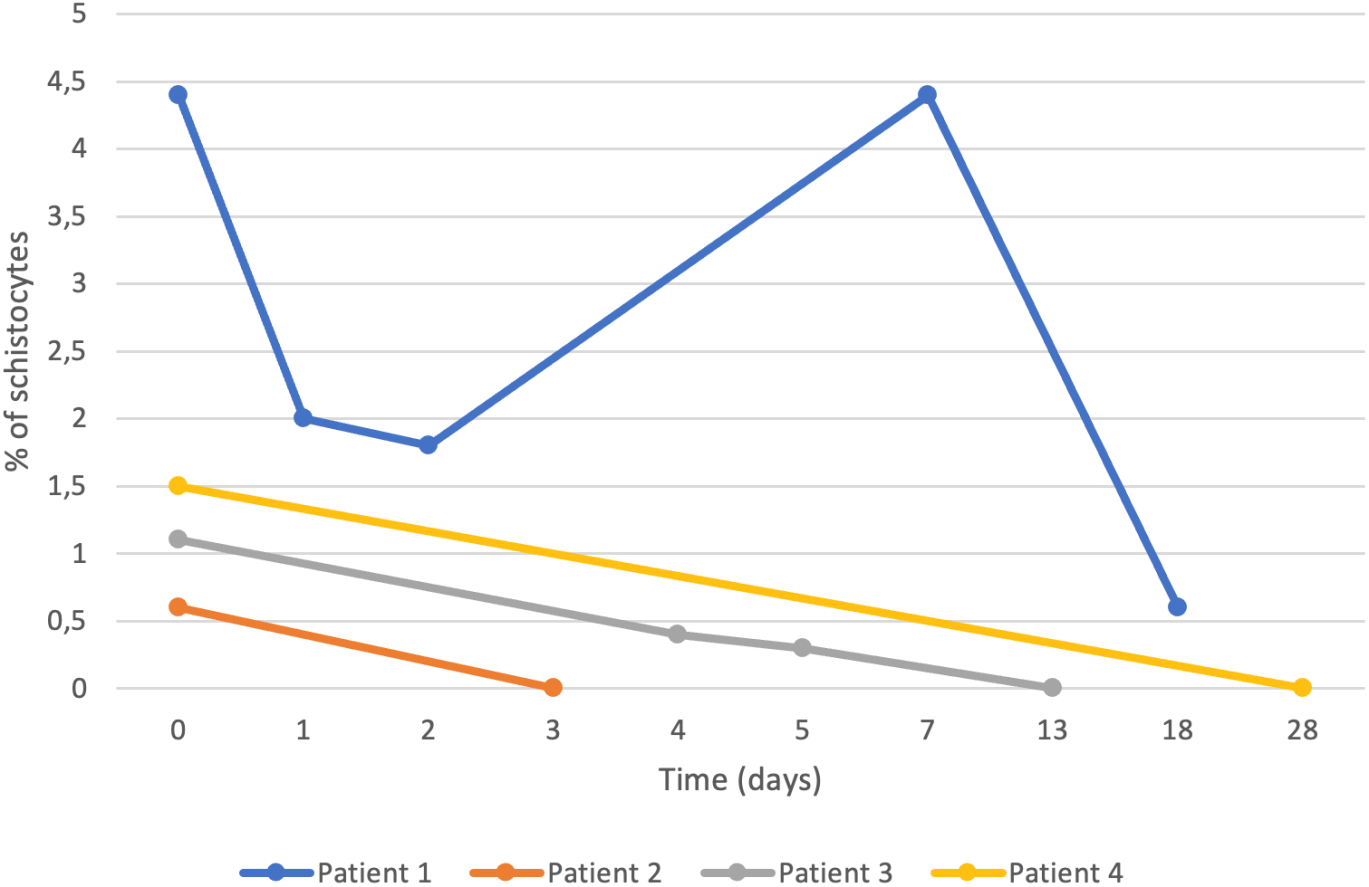
Change in schistocyte levels after discontinuation of Dapsone.

Dapsone was discontinued in 9 patients in group A and in 1 patient in group B due to anaemia, schistocytes and/or methaemoglobinaemia. Dapsone discontinuation was associated with a decrease in the number of schistocytes in patients with a follow-up blood smear (Fig 2). Schistocytes persisted for up to 5–18 days in two patients depending on initial schistocyte levels. Discontinuation of treatment resulted in an increase in haemoglobin: + 2.4g/dL (+1.4; + 3.5) after 1 month in group A.

## Discussion

This study reports the presence of schistocytes associated with low haptoglobin, high LDH, high reticulocytosis and anaemia in patients treated with dapsone. Our findings suggest a microangiopathic hemolytic pattern that may be indicative of thrombotic microangiopathy (TMA), although insufficient data are available to establish this diagnosis; no target organ damage was observed, and no histological data were available. Schistocytosis disappeared upon discontinuation of dapsone.

Oxidative haemolysis is classically described in dapsone treatment [19,20], but the presence of schistocytes has rarely been reported. Naik et al reported cases of dapsone-associated haemolytic anaemia in lung allograft recipients, with schistocytes on blood smears reported in 5 out of 10 patients [12], though in the setting of xenograft which is a known cause of TMA. Lor et al described dapsone-associated anaemia in heart transplant recipients. The presence of schistocytes was observed in 1 out of 8 patients. Anaemia improved after discontinuation of treatment, but changes in schistocyte count were not reported [21]. The only ischemic organ damage associated with dapsone and schistocytes in the peer-reviewed literature is retinal ischemic damage, which was reported in a patient treated for bullous dermatitis who underwent acute dapsone poisoning with haemolytic anaemia, schistocytes (2–5%) and massive methaemoglobinaemia (32%) [13]. Another case report described an acute confusional state in a 36-year-old patient treated with corticosteroids for chronic interstitial nephritis and dapsone for prophylaxis. Biology revealed hypoxia with 11% methaemoglobinaemia and thrombotic microangiopathy pattern (haemolytic anaemia with schistocytes, thrombocytopenia and acute renal failure). A favourable outcome was observed after discontinuing dapsone and administering methylene blue [22]. Dapsone-associated schistocytes have also been reported in patients followed for relapsing polychondritis [23] acute lymphoblastic leukaemia [24] and idiopathic urticaria [25]. Thus, the presence of schistocytes has been reported in several pathologies, emphasizing the attribution of schistocytosis to treatment with dapsone rather than the underlying condition.

Furthermore, in the French pharmacovigilance database, 12 cases of dapsone-induced haemolytic anaemia in which the presence of schistocytes was mentioned were identified. Median time to identification of schistocytes was 24 days after dapsone start (IQR 17;40 days). The value of schistocytes was reported in 11 cases and ranged from 1 to 15%. No renal failure or target organ damage was reported. In 4 cases, a decrease in schistocytes after discontinuation was observed and in 1 case a decrease was observed despite continuing treatment (no information on the outcome in 7 cases). One of the patients reported with TMA on dapsone presented with dyspnoea, psychomotor slowing, and schistocytosis up to 15%. He received plasma exchange for strong suspicion of TMA. Methaemoglobinaemia was not reported, and extensive evaluation for primary or secondary TMA was negative, with no target organ damage detected, including on brain MRI.

In the setting of thrombocytopenia and haemolytic anaemia, schistocytosis is strongly evocative of TMA, which is a medical emergency. Our data show that patients treated with dapsone—including those with ITP and concomitant thrombocytopenia—develop a characteristic TMA-like laboratory profile. Schistocytosis in patients treated with dapsone is of special concern as haemolytic anaemia is almost constant. In this series, the discovery of schistocytes sometimes had significant clinical consequences such as prompted or prolonged hospitalization and led to the discontinuation of dapsone in most patients. In sharp contrast with TMAs, none of our patients had detectable ischemic organ damage, including those patients with elevated schistocytosis who underwent a complete TMA workout.

The relatively high incidence of low-grade schistocytosis in the general population (estimated mean reported in studies ranges between 0.05% and 0.15%) raises the possibility of a fortuitous association with dapsone therapy [26,27]. However, the significant ratio of schistocytes to red blood cells in several patients and the regression of schistocytosis after discontinuation of dapsone, along with the absence of schistocytes before the introduction of dapsone in others, plead for the imputability of dapsone. The pathophysiological relationship between dapsone treatment and schistocytes is unclear. Given the report of some cases of schistocytosis associated with elevated methaemoglobinaemia, we wondered whether methaemoglobinaemia itself could induce a membrane rupture mimicking mechanical haemolysis. Methaemoglobinaemia is a dose-dependent side effect often described in the literature [28], especially in cases of dapsone overdose [29]. This could be supported by methaemoglobinaemia being higher in group A in this study. Haemolysis and methaemoglobinaemia are the consequence of dapsone toxic hydroxylamine metabolites responsible for an alteration of the pentose cycle within red blood cells [30]. This metabolic pathway of dapsone is carried out by the hepatocytes via cytochrome P450. Thus, we hypothesize that genetic polymorphisms of these cytochromes or a drug interaction could influence this pathway and the toxicity of dapsone [31]. Another pathophysiological hypothesis would be that dapsone might be responsible for a secondary TMA. Bian et al suggested that dapsone hydroxylamine could increase thrombotic risks by inducing the procoagulant activity of red blood cells in an *in vitro* study and in a murine model [32]. The mechanisms, which have not been fully elucidated, could be an impairment of the phospholipid membrane, an increase in thrombin production, self-aggregation of red blood cells and their adhesion to endothelial cells.

Dapsone-triggered schistocytosis, especially in the setting of ITP, raises the crucial question of whether schistocytes are pathogenic or not, with two critical implications. First, in which cases should the appearance of schistocytes under dapsone lead to an emergency workup to rule out TMA? In our series, no patients suffered from complications of schistocytosis, despite an extensive workup in some of them. In the literature, we retrieved one case of retinal ischemia, but concomitant with severe dapsone overdose [13]. In the pharmacovigilance cases, one patient was treated for presumed TMA due to psychomotor slowing, but methaemoglobinaemia was not provided and no ischemic target organ damage could be demonstrated – namely he had a normal brain MRI despite neurological presentation. To date, no definite cases of TMA related to dapsone have been reported at therapeutic dosage. Second, should the discovery of schistocytes on dapsone therapy lead to systematic decrease or discontinuation of this treatment, which was the case in most patients of our series? At this time, no scientific data could help answer this question.

Our study has strengths. We conducted the first retrospective study specifically examining the association of schistocytes with dapsone treatment. All blood smears were re-examined by a single biologist in a blinded manner to reduce inter-operator variability. The findings were generally consistent with the initial analyses. We supported our results by using the national pharmacovigilance database. However, our study also has several limitations. Blood smears are not routinely performed in the follow-up of dapsone treatment, which is why we could include only 19 patients despite an extensive review. Our study was therefore underpowered to determine statistical significance. Its retrospective design occasioned missing data. For example, haemolytic anaemia workups were not always complete, and we were only able to accurately estimate the time to disappearance of schistocytes after cessation of treatment at an approximate interval of 3–28 days. G6PD testing was not performed for all patients, or the data were missing from the patients’ medical records, which raises the possibility that some reported patients may have an undiagnosed G6PD deficiency. However, drug-induced hemolytic anemias in the context of G6PD deficiency are usually not accompanied by schistocytosis, as the hemolysis is oxidative rather than mechanical. We were not able to retrieve or demonstrate any ischemic organ damage in any of the affected patients in our hospital or in the French database.

## Conclusion

Overall, we report that schistocytosis onset can be associated with dapsone treatment. Thus, it can lead to inappropriate complementary workup, hospitalization or inappropriate invasive treatment, and treatment modifications. Even though none of the patients included demonstrated any organ complications, the clinical meaning of schistocytosis during dapsone treatment remains unclear, and discontinuation or tapering of this drug should be discussed. In the absence of sufficient data, the presence of schistocytes should primarily prompt the exclusion of thrombotic microangiopathy, either primary or secondary. Our data suggest that clinical and biological monitoring of patients treated with dapsone is relevant. Prospective studies in patients on chronic dapsone treatment should provide further insight into the clinical implications of this condition.

## Supporting information

S1 Table Clinical and biological data of all patients in group A. This table summarizes all clinical and laboratory characteristics of group A, including the indication for dapsone therapy, patients’ medical history, the time interval between treatment initiation and the appearance of schistocytes, the presence of cytopenias, evidence of hemolysis, renal function, and the possible occurrence of methemoglobinemia.
(DOCX)
