# Supplementary material for: Dapsone and schistocytes: Thrombotic microangiopathy or not?
Source: PLoS One. 2026 May 22;21(5):e0349570. doi: 10.1371/journal.pone.0349570 (PMC13196926; doi:10.1371/journal.pone.0349570)
Supplement: S1 Table — This table summarizes all clinical and laboratory characteristics of group A, including the indication for dapsone therapy, patients’ medical history, the time interval between treatment initiation and the appearance of schistocytes, the presence of cytopenias, evidence of hemolysis, renal function, and the possible occurrence of methemoglobinemia. (DOCX) [file pone.0349570.s001.docx]

*S1 Table : Clinical and biological data of all patients in group A*

| **Patient** | **Pathology^1^** | **Relevant medical history** | **Treatments^2^** | **Delay (days)^3^** | **Schistocytes (%)** | **Hemoglobin**  **(g/dl)** | **Platelet (G/L)** | **Haptoglobin (g/L)** | **LDH (U/L)** | **Créatinine**  **(μmol/L)** | **MetHb**  **(%)** |
| --- | --- | --- | --- | --- | --- | --- | --- | --- | --- | --- | --- |
| **1** | ITP | Ischemic heart disease (coronary artery bypass grafting), mitral bioprosthesis, atrial fibrillation, pacemaker, hypertension, diabetes | / | 21 | 4.4 | 8 | 141 | <0.3 | 612 | 101 | 5.8 |
| **2** | Mucous membrane pemphigoid | Hypertension, diabetes | / | 407 | 2.0 | 8.4 | 128 | <0.3 | 542 | 166 | 1.6 |
| **3** | Bullous pemphigoid | Ischemic heart disease (stenting and bypass grafting), peripheral arterial disease, diabetes, hypertension, right femoral angioplasty, left femoral tripod endarterectomy, left transfemoral amputation | Simvastatine  (> 6 years) | 15 | 1.5 | 7.8 | 264 | 1.3 | 557 | 127 | 5.9 |
| **4** | ITP | / | / | 29 | 1.1 | 12.3 | 34 | <0.3 | 261 | 82 | / |
| **5** | Lupus^4^ | / | / | 367 | 1.5 | 10.5 | 44 | <0.3 | 584 | 71 | 7.7 |
| **6** | ITP | Follicular lymphoma in partial remission, Lieberkühnian adenocarcinoma in remission, discovery of suspicious pulmonary nodules | / | 66 | 3.5 | 12.5 | 330 | <0.3 | 671 | 84 | 4.6 |
| **7** | / | Multiple sclerosis | / | 6 | 0.4 | 12.6 | 199 | <0.3 | 239 | 92.5 | 15.6 |
| **8** | ITP | Gestational diabetes, gestational hypertension, preeclampsia^5^ (resolved after delivery) | Progesterone | 40 | 0.2 | 12.4 | 7 | <0.3 | 297 | 58 | / |
| **9** | Pemphigus vulgaris | Hypertension, hypertrophic cardiomyopathy | / | 150 | 0.6 | 9.8 | 233 | <0.3 | 600 | 118 | 1.5 |
| **10** | ITP | Ischemic stroke, epilepsy, hypertension, pulmonary embolism, left papillary carcinoma with partial nephrectomy (in remission) | / | 114 | 0.2 | 10.6 | 60 | / | / | 81 | 1.3 |
| ^1^ The recorded pathology corresponds to the reported indication for initiating the treatment. In one patient, the indication was not clearly specified.  ^2^ Only treatments described as causing thrombotic microangiopathy have been listed.  ^3^ The delay corresponds to the interval between the first day of treatment and the performance of the blood smear.  ^4^ In the context of lupus pathology, a complement assay was performed concurrently (C3 0.83 g/L, C4 0.16 g/L).  ^5^ The preeclampsia was associated with the presence of schistocytes. Following the episode, the patient underwent a follow-up blood smear, which showed no schistocytes (prior to the initiation of dapsone treatment).  No histological analyses, including renal biopsies, were available for any of the patients.  ADAMTS13 assay was performed in only 4 patients, and no significant deficiency was observed  MetHb : methaemoglobin ; LDH : lactate dehydrogenase. | | | | | | | | | | | |

**REFERENCES**

Mazzierli T, Allegretta F, Maffini E, Allinovi M. Drug-induced thrombotic microangiopathy: An updated review of causative drugs, pathophysiology, and management. Front Pharmacol. 2023;13:1088031. Published 2023 Jan 9. doi:10.3389/fphar.2022.1088031
